# Developing a multidisciplinary pediatric cancer predisposition service at a mid-sized children’s hospital

**DOI:** 10.3389/fonc.2026.1794874

**Published:** 2026-05-11

**Authors:** Amanda M. Harrington, John August D’Orazio

**Affiliations:** 1 Department of Pediatrics, University of Kentucky, Lexington, KY, United States; 2 DanceBlue Pediatric Hematology/Oncology Clinic, The Markey Cancer Center, University of Kentucky College of Medicine, University of Kentucky, Lexington, KY, United States

**Keywords:** cancer genetics, cancer predisposition, clinical research, community engagement, Li-Fraumeni syndrome, pediatric oncology

## Abstract

Roughly one in ten children and young adults diagnosed with cancer has an underlying genetic variant that may explain his/her malignancy. It is critical to identify patients with cancer predisposition syndromes (CPSs) to provide personalized cancer treatment, surveillance, counseling, psychosocial support, and in some cases, risk-reducing interventions to maximize long-term outcomes. Over the past five years, we developed a comprehensive pediatric CPS program at our institution. We engaged our institutional adult cancer center for clinical cancer genetic counseling services and needed research shared resource facilities. We also initiated a clinical study called “Project Inherited Cancer Risk” (PICR) to identify pediatric oncology patients affected by CPSs through universal germline NextGen sequencing of a known panel of CPS genes. Finally, we have engaged the CPS community to learn how we can better serve them. Here, we describe the rationale, process, challenges and opportunities of developing a multidisciplinary CPS clinical service in a medium-sized academic pediatric oncology center.

## Background/introduction

Cancer predisposition syndromes (CPSs) are caused by germline loss-of-function pathogenic variants in tumor suppressor genes or gain-of-function pathogenic variants in oncogenes that result in an individual having an increased chance of developing cancer compared to the risk of unaffected persons. Two landmark studies suggest that roughly one in ten newly diagnosed pediatric oncology patients has an identifiable inherited cancer risk factor (1, 2). A recent report studying the COG MATCH trial for patients with treatment-refractory solid tumors, non-Hodgkin lymphomas, or histiocytic disorders - found germline cancer predisposing genetic variants in 73 of 1,167 (6.3%) samples (3), while another study of over 750 children with solid tumors found germline variants in 18% of patients, with 34% of patients having germline pathogenic variants that were unexpected given their diagnosis and clinical history (4). Taken together, these studies suggest that approximately one in ten young people who are diagnosed with cancer have inherited a gene variant that predisposes them to developing cancer. With approximately 16,000 children diagnosed with cancer in the US each year, more than 1,500 of them will have an underlying CPS, many of which carry significant clinical care implications beyond the treatment of their initial cancer. As survival improves for young people with cancer, identifying a potential CPS can help guide follow up care, may alter their future cancer surveillance, and is also important for their relatives (5).

The timely recognition of pediatric CPS patients is a crucial component of comprehensive medical care. Children affected by a CPS are usually identified in one of four general ways (1): the child is diagnosed with a malignancy, has clinical signs of an inherited CPS and is genetically tested for syndromes compatible with the child’s diagnosis, family cancer history and/or clinical findings (2), the child is tested for a specific gene variant during cascade testing after another family member is identified to have a CPS (3), genetic testing through clinical studies offering germline sequencing, and (4) somatic clinical testing identifies a pathogenic variant that is then confirmed germline. Our institution identifies CPS patients through each of these mechanisms.

## Benefits of identifying CPS

A rich literature supports the identification and personalized management of patients with inherited cancer syndromes (reviewed in (6–10)). Villani, et al. found improved overall survival in patients with Li-Fraumeni Syndrome who underwent cancer surveillance (11). The authors concluded that “early tumor detection through surveillance is associated with improved long-term survival” … and that “incorporation of this approach into clinical management of these patients should be considered” (12). Optimal management of the pediatric or young adult patient with a cancer syndrome involves specialized understanding of what cancers the patient is at risk for, what co-morbidities exist because of the genetic defect, and at what ages possible malignancies and other syndrome-related complications are likely to arise. As an almost universal rule, malignancies are more easily treated and have a more favorable prognosis when caught early, emphasizing the utility for careful surveillance of, and in some cases, cancer-preventive interventions for the at-risk patient.

## Cancer predisposition clinic

Recognizing that the oncologic care aspects of pediatric patients with CPS may fall beyond the scope of primary care providers, we created the DanceBlue Pediatric Cancer Predisposition Clinic in 2020 out of the growing need to offer subspecialized care to patients and families affected by hereditary cancer in our region. This clinic is housed within our pediatric hematology and oncology clinic, which is a hospital-based clinic, and funded by clinical revenue. We started with a physician, a nurse navigator and a cancer genetic counselor meeting patients during a half-clinic day each month. The clinic was developed to care for CPS patients identified clinically or by referrals from cancer genetic counselors, typically after cascade testing of family members of adult patients identified to have a CPS or if there is a suspicion of a CPS in the patient’s family. For patients suspected of having a CPS who have not had definitive genetic testing, the workflow typically begins with patients being referred to a cancer genetic counselor to review clinical and family medical history and to obtain genetic diagnostic testing.

After a child, adolescent or young adults is definitively identified to have a germline CPS, then the patient is scheduled to be evaluated in our CPS clinic where an oncologist specializing in inherited cancer then meets with the patient and family to review their cancer risk and discuss surveillance options based on the specific inherited cancer syndrome. Several groups have published surveillance recommendations, including the National Comprehensive Cancer Network (NCCN), the American Association of Cancer Research (AACR) Childhood Cancer Predisposition Workshops, as well as individual disease-specific societies (5, 13–28). We work with patients and families to incorporate and consolidate up-to-date recommendations for cancer surveillance and develop personalized management plans that are in alignment with patient and family goals. Over the past 5 years, we have seen steady growth of this clinic through increased disease recognition and referrals and have now expanded to 3 half-day clinic sessions per month and see nearly 100 patients with cancer predisposition annually. As a small-to-medium sized program, the model for our physicians is to be general pediatric hematologists/oncologists (e.g. for ward coverage), but also to be able to specialize in a given area of expertise in outpatient clinic. The provider who oversees our CPS clinic devotes roughly 30% of her clinical service time to the CPS service. We have relied on cancer genetic counselors from the Markey Cancer Center (our adult cancer center) and have one embedded in our CPS clinic, which meets two-to-three half days per month, to counsel patients and discuss results and testing with families. The cancer genetics counselor is also available to coordinate cascade genetic testing, and those discussions typically happen in the context of a CPS clinic visit.

**Figure 1:**
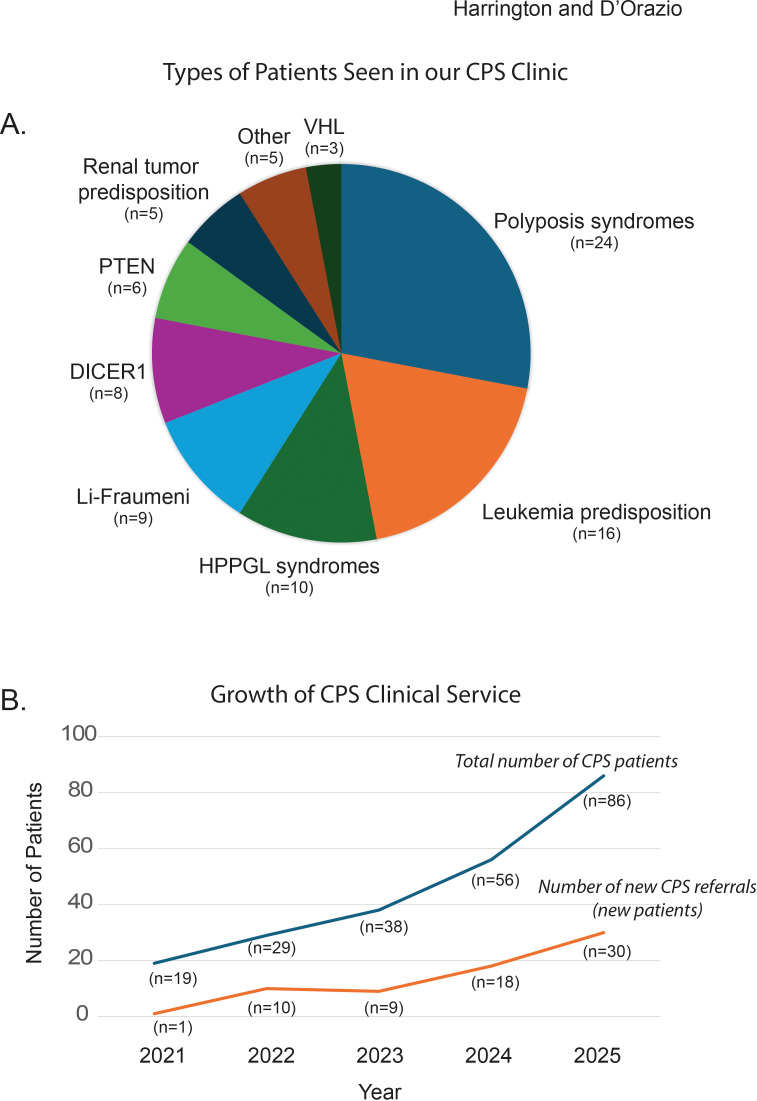
**(A)** Types of patients seen in our current CPS clinic (by cancer predisposition type). Please note that VHL indicates Von Hippel-Lindau and HPPGL indicates Hereditary Paraganglioma-Pheochromocytoma Syndrome. We have indicated current numbers of patients of each type by “n”. **(B)** Growth of our CPS clinical service over time since it started in 2020. Shown are the numbers of individual CPS patients (blue line) and new patient referrals (orange line), with the numbers per year indicated by “n”.

Our clinic cares for patients with a variety of CPSs with polyposis syndromes, leukemia predisposition syndromes and Li-Fraumeni patients making up the majority (**Figure 1**). As many of these syndromes require multidisciplinary care, we strive to reduce the burden of medical care as much as possible by coordinating appointments with subspecialty providers. Patients needing imaging will typically do so in the mornings and then report to CPS clinic in the afternoon on the same day to review any findings. We have also been able to add a dedicated psychologist to our hematology/oncology clinic and extend these services to the CPS clinic. Patients and parents can meet with our psychologist to discuss psychosocial needs. We also have school liaisons to help those undergoing frequent medical visits to ensure a smooth connection between the hospital and the school system to ensure these patients are not falling behind. Our goal is to provide a medical home and source of broader medical care coordination for the patients and families affected by cancer predisposition. An additional method we have employed to help identify patients with CPS is through a clinical study called Project Inherited Cancer Risk (PICR). This study is intended to identify patients with CPS by offering germline focused exome sequencing to all patients with a pediatric cancer diagnosis.

## Project inherited cancer risk

To date, the identification of children and young adults with CPSs has traditionally relied on clinical clues. However, it is estimated that roughly half of patients affected by an inherited cancer syndrome fail to be identified by conventional clinical measures (8, 29, 30). To address this, we developed “Project Inherited Cancer Risk” (PICR)—a clinical study offering focused exome germline testing to all patients with a new or previous diagnosis of pediatric cancer. We felt that the knowledge gained by understanding our patients’ inherited cancer risk would be imperative to their survivorship and future cancer journey, and therefore the development of this clinical opportunity would help us offer more comprehensive care to our patients.

Reasoning that sequencing costs would likely be lower if done in-house, we worked with personnel from our institution’s Oncogenomics Shared Resource Facility (SRF), which is a core facility supported by our institutional (Markey Cancer Center) Cancer Center Support Grant (CCSG). The Oncogenomics facility is embedded in our healthcare system’s pathology department and provides cutting-edge genomic and molecular technology services including NextGen focused exome sequencing. We developed a panel of 128 genes in which the literature supported a link between variants and malignancy in children, adolescents and young adults (**Table 1**). Next, we negotiated with the healthcare system to reduce the cost of NextGen sequencing for PICR. We then engaged our pediatric hematology/oncology division’s clinical research office as well as the clinical pathology laboratory to develop workflow and standard operating procedures for PICR. We met with leaders from several CCSG-supported SRFs to develop a protocol for specimen handling and analysis. The Biospecimen Procurement and Translational Pathology SRF collects, processes, annotates and distributes clinical PICR samples. The Oncogenomics SRF provides genomic and molecular technology services in the form of NextGen focused exome sequencing, as well as data curation and development of CLIA-certified reports that are entered into the patient electronic medical record. The custom PICR gene sequencing panel is derived from a Medical Exome Sequencing (MES) panel. Specifically, the pass filter for percentage of reads >Q30 is **≥** 90%, and minimum reads for variant calling is **≥** 20x. Exonic and adjacent intronic regions (± 50bp) are evaluated and variants are classified according to American College of Medical Genetics and Genomics (ACMG) variant classification guidelines. Only pathogenic/likely pathogenic variants are reported, and results are uploaded into the electronic medical record as a lab test result, with an embargo of three days so that our team can personally discuss results with patients identified to harbor a variant in the cancer gene panel. The Cancer Research Informatics SRF organizes and provides a home for the project’s data, and the Biostatistics and Bioinformatics SRF offers statistical expertise to ensure rigor and enhance the execution of the clinical study. PICR was opened in August 2021 and has been accruing patients since then.

**Table 1:** Cancer-related genes on the PICR panel.

*AIP*	*DIS3L2*	*GREM1*	*PDGFRA*	*SDHB*
*ALK*	*DKC1*	*HAX1*	*PHOX2B*	*SDHC*
*APC*	*DNMT3A*	*HOXB13*	*PMS2*	*SDHD*
*ASXL1*	*EGFR*	*HRAS*	*POLD1*	*SH2B3*
*ATM*	*ELANE*	*IKZF1*	*POLE*	*SMAD4*
*AXIN2*	*EPCAM*	*JAK2*	*POT1*	*SMARCA4*
*BAP1*	*ETV6*	*KIT*	*PPM1D*	*SMARCB1*
*BLM*	*EXT1*	*KRAS*	*PRKAR1A*	*SMARCE1*
*BMPR1A*	*EXT2*	*LZTR1*	*PTCH1*	*SRP72*
*BRCA1*	*FANCA*	*MAX*	*PTEN*	*STK11*
*BRCA2*	*FANCB*	*MBD4*	*PTPN11*	*SUFU*
*BRIP1*	*FANCC*	*MECOM*	*RAD51C*	*TERC*
*BUB1B*	*FANCD2*	*MEN1*	*RAD51D*	*TERT*
*CBL*	*FANCE*	*MET*	*RB1*	*TET2*
*CDC73*	*FANCF*	*MITF*	*RECQL4*	*TINF2*
*CDH1*	*FANCG*	*MLH1*	*REST*	*TMEM127*
*CDK4*	*FANCI*	*MSH2*	*RET*	*TP53*
*CDKN1B*	*FANCL*	*MSH3*	*RPS19*	*TRIM28*
*CDKN1C*	*FANCM*	*MSH6*	*RPS20*	*TRIP13*
*CDKn2A*	*FH*	*MUTYH*	*RTEL1*	*TSC1*
*CEBPA*	*FLCN*	*NBN*	*RUNX1*	*TSC2*
*CEP57*	*G6PC301*	*NF1*	*SAMD9*	*VHL*
*CHEK2*	*GATA1*	*NF2*	*SAMD9L*	*WRN*
*CTNNA1*	*GATA2*	*NTHL1*	*SBDS*	*WT1*
*DDX41*	*GFI1*	*PALB2*	*SDHA*	
*DICER1*	*GPC3*	*PAX5*	*SDHAF2*	

We configured PICR eligibility to include anyone with a personal history of pediatric cancer, and our divisional clinical research office identifies patients eligible for the study. The nurses and clinical research associates (CRAs) in that office approach a given patient’s treating oncologist and ask them to consider offering PICR to the patient. Each of our oncology providers is supportive of PICR, so in practice, it becomes a matter of timing for when to approach patients and families based on clinical status and opportunity. For solid tumor or central nervous system tumor patients, a sample of non-neutropenic blood is adequate, typically collected in our outpatient clinic or on the inpatient unit. For leukemia patients, our practice had been to collect blood or saliva after confirmed remission with an M1 marrow and negative minimal residual disease (< 0.01% by flow cytometry), but because of concerns about peripheral blood contamination with malignant cells, somatic mosaicism, or somatic genetic reversion events, we now prefer cultured fibroblasts taken from a small skin biopsy performed at the time of central line insertion or other sedated procedure. The consent/assent process is typically done in two stages, with the oncologist describing and offering the study, and a clinical research nurse or CRA following up with more information about PICR and the option of meeting a cancer genetic counselor should the family be interested. We are currently in the process of hiring a dedicated genetic counselor who will be housed in our pediatric oncology clinic and envision that this person will be integrally involved in the consenting and reporting processes of PICR. Neither the family nor their insurance will be charged for PICR testing, and patients are not obligated to participate in the study. The PICR project reports pathogenic or likely pathogenic results back to the patient’s medical record, and the study PI also contacts patients with results (letter for negative findings, telephone call or face-to-face discussion for positive findings). The consent form states “We will inform you about your results for the clinically-recognized cancer genes. Only mutations that are known to cause increased cancer risk will be reported for clinical management purposes.” To date, the project has accrued more than 300 patients and has served as a referral source for our CPS service by identifying patients (and family members) with CPS. We find that our accrual is >90% of patients who are offered the study. Overall, PICR has identified pathogenic or likely pathogenic variants in 10.2% of study participants. Patients identified with a CPS either through PICR or other means (e.g. by cancer genetic counselors) are then referred to our CPS clinic.

## Pediatric germline molecular tumor board

Optimal management of the cancer-predisposed patient demands coordinated and multidisciplinary care between medical professionals. We developed a multidisciplinary germline tumor board conference composed of pediatric oncologists, cancer genetic counselors, pharmacists, pathologists, bioethicists, interested cancer researchers, and representatives from the Markey Cancer Center SRFs to review sequencing findings from our PICR project. This group has convened to review patients with positive “hits” or concerning cancer gene variants and discusses them within the context of the patient’s clinical and family history. The team then formulates recommendations for management based on the current understanding of relevant gene alterations and clinical status. Identifying one family member with a cancer syndrome immediately raises the possibility that other members of the kindred may be affected, and there are important ethical and legal considerations to acknowledge regarding assent, consent and diagnosis. For all these reasons, we routinely refer families in which a pathogenic or likely pathogenic alteration in a CPS gene has been identified to cancer genetic counselors for further exploration of results.

## Engaging the community

Realizing that the care of CPS patients is multidisciplinary, and wanting to understand how we could better serve the needs of our CPS community, we took advantage of an internal “Partnership Planning” funding mechanism offered by the Markey Cancer Center’s Community Impact Office to convene an advisory council made up of CPS patients, their caregivers and other stakeholders in the CPS community. This group, which met quarterly for two years, was updated about our CPS clinical program as well as research projects derived from PICR. With this council’s input, we identified a need for more psychosocial resources to support patients and families affected by hereditary cancer. One issue that the advisory council brought up to us was the need to understand and address psychosocial aspects associated with a child with a CPS diagnosis. Specifically, having a child diagnosed with a CPS is associated with stress about cancer risk, anxiety about surveillance testing (e.g. “will this be the time they find a cancer in my child?”), and genetic guilt for the parent identified to be a carrier of the CPS variant (31–33). Through direct interviews with our CPS advisory group, we identified “pinch points” when psychosocial support is especially needed, such as on days when surveillance imaging is reviewed. We leveraged these insights to hire a psychologist to be embedded into our pediatric hematology/oncology clinic to help address this need. The community group also provided input that helped our CPS team host two annual meetings on cancer predisposition that attracted CPS patients, caregivers, providers, researchers and other community stakeholders to half-day symposia that reviewed clinical CPS, highlighted relevant research being done at our institution and featured patient testimonials and panel discussions. Finally, the CPS advisory panel prompted us to investigate the psychosocial stressors and coping responses in our CPS community further in the form of a mixed methods study, supported by a Community-Engaged Research Pilot grant from the Markey Cancer Center’s Community Impact Office.

## Supporting CPS efforts

We developed a pediatric CPS service by leveraging expertise in pediatric oncology with programs and services offered by the NCI-designated comprehensive cancer center at our institution, including the cancer genetic counseling program, several NCI-supported shared resource facilities, and funding opportunities provided as part of the NCI-funded cancer center support grant (CCSG). In addition, our philanthropy offices were able to identify donors who generously provided funds to support sequencing and other costs related to PICR. Finally, we were able to secure state funding from the Kentucky Pediatric Cancer Research Trust Fund (KPCRTF) not only to cover sequencing costs but also to develop and maintain the pediatric germline molecular tumor board, to expand PICR into a statewide program (offered at Norton Children’s Hospital at the University of Louisville), and to fund investigative research spawned from PICR.

## Challenges

The CPS program at our institution is flourishing and growing, but there have been several challenges. From a programmatic standpoint, it took time and many meetings to convince hospital administration to provide sequencing and biobanking services at reduced rates. We also acknowledge that CPS testing is an evolving entity with more genes added to the cancer gene panel for testing over time, which translates to many meetings with the Oncogenomics sequencing staff and amendments to our IRB protocol. To avoid the situation wherein genes that were not listed on the consent/assent documents that patients or their families signed, we developed a generalized IRB-approved statement in the consent/assent documents with a weblink to genomic human subjects research from the National Human Genome Research Institute instead of listing specific genes being tested to avoid risking discordance between genes the patient agrees to have tested with those issued in their clinical report.

## Life-long CPS care

A major challenge at our institution is the limited number of adult clinics with expertise to manage patients with CPS once they “age out” of our pediatric CPS service. At our NCI-designated comprehensive cancer center, there is not a dedicated provider doing comprehensive cancer screening for adults with CPSs except for specific breast/ovarian and colorectal cancer syndromes. We have discussed the opportunity to develop a CPS transition service with our adult counterparts from the cancer center. Li-Fraumeni syndrome (LFS), for example, is a CPS in which affected patients may develop multiple types of cancer throughout their lifetime. Aside from increased screening in the breast/ovarian or colorectal cancer clinics, LFS patients must find other providers for more generalized cancer screening, and there is no one clinic to provide a comprehensive CPS medical home in the cancer center for such patients. LFS patients have one of the most rigorous and well-studied surveillance regimens which require doctor visits and cancer surveillance multiple times per year. Due to lack of available comprehensive CPS providers in our institution’s adult oncology practice, we have continued to care and offer cancer surveillance for adult CPS patients, involving adult surgical and medical specialists rather than pediatric subspecialty colleagues when needed. Our goal is to work together with our adult oncology institutional partners to develop transition programs for CPS patients that impact health into adulthood.

As we continue to learn more in this field, we recognize that cancer screening recommendations and variant pathogenicity may change over time. For this reason, patients with a CPS are recommended to stay in touch with cancer genetics at least every 2–3 years to ensure that the latest medical recommendations are communicated with the families. Additionally, as the pediatric patient matures into adulthood, we advise our patients to discuss reproductive options and to consider partner testing as needed. We appreciate that these conversations become even more important as adolescent patients grow and develop autonomy for their own medical decision making.

## Conclusions

The pace at which our CPS programs have evolved has been remarkable, from one to now three half-day clinics needed per month to meet the needs and accommodate both new referrals and surveillance visits. Clinical volumes of the pediatric, adolescent and young adult CPS service are now at nearly 100 families cared for in our clinic each year. Our catchment area spans Eastern Kentucky, including Appalachia—an area known for its increased incidence of malignancies. We are uniquely positioned within central Kentucky to provide care for many patients in Eastern Kentucky and surrounding areas.

Specialized care centers for CPS patients have historically been available at larger academic institutions. Golisano Children’s at UK is considered a medium-sized cancer program, seeing between 75–90 new cancer diagnoses each year. By building a multidisciplinary CPS clinic and leveraging institutional strengths of our comprehensive cancer center, we are pleased to offer a medical home for young CPS patients in Kentucky who would otherwise need to go out of state for their cancer predisposition management. Being a children’s hospital within a larger adult-based medical center enables us to coordinate patient care across a continuum of ages to ensure safe and successful transitions as our patients “age out” of pediatric subspecialty care. From patient testimonials, we are certain that our efforts are of benefit to many children and families affected by CPS in our catchment area. Moreover, basic, translational, and psychosocial research projects have blossomed from our CPS efforts, and we have engaged the CPS community by involving them and acting on their advice and suggestions to keep our programs vibrant and relevant. Together, our aim is to provide a multidisciplinary home for pediatric CPS patients and their families to offer them comprehensive medical and psychosocial care.

## Data Availability

The original contributions presented in the study are included in the article/supplementary material. Further inquiries can be directed to the corresponding author.
